# The influence of complex classroom noise on auditory selective attention

**DOI:** 10.1038/s41598-025-18232-2

**Published:** 2025-09-25

**Authors:** Carolin Breuer, Robert Josef Schmitt, Larissa Leist, Stephan Fremerey, Alexander Raake, Maria Klatte, Janina Fels

**Affiliations:** 1 Institute for Hearing Technology and Acoustics, RWTH Aachen University, 52074 Aachen, Germany; 2 Cognitive and Developmental Psychology, RPTU Kaiserslautern-Landau, 67663 Kaiserslautern, Germany; 3 Audiovisual Technology Group, Technische Universität Ilmenau, 98693 Ilmenau, Germany; 4 Biological Psychology, RPTU Kaiserslautern-Landau, 76829 Landau, Germany; 5 Chair for Signal Processing in Communications Engineering, RWTH Aachen University, 52074 Aachen, Germany

**Keywords:** Auditory selective attention, Classroom, Noise, Binaural hearing, Complex acoustic environment, Virtual reality, Speech noise, Human behaviour, Acoustics

## Abstract

Recent efforts to mimic real-life situations in experiments aim to improve the ecological validity of research. Acoustically, this often involves using binaural reproduction to create realistic environments. While studies have shown that simplified acoustic presentations, such as white noise, affect children’s auditory selective attention without impacting adults, other research suggests that these effects might be overestimated in monaural scenarios. This underscores the need for more realistic approaches. The current study introduced spatialized, non-stationary classroom noise with and without speech, comparing it to white noise and a silent baseline in a child-appropriate experiment on auditory selective attention in a virtual reality classroom. Findings from adult participants, who were tested to validate the paradigm, indicated significantly higher error rates for realistic classroom noise compared to white noise and silence. Trials with intelligible speech as a distractor were particularly disruptive to auditory attention. Participants also reported higher mental demand, perceived effort, and task difficulty with complex noise types. These results emphasize the importance of using complex noise scenarios in auditory cognition research to draw conclusions applicable to real-life situations. This approach provides a more accurate understanding of how realistic classroom noise affects auditory selective attention.

## Introduction

When thinking about acoustically challenging everyday life scenarios, cocktail party situations as first introduced by Colin Cherry in 1953^1^ are very common. This term describes a variety of situations where the listener is faced with multiple competing sound sources, while concentrating on one specific sound source. In a cocktail party, the listener would focus on the person they are talking to while filtering the surrounding sounds such as other conversations or ambient music in the background. This task can become even more challenging, when attending to more than one sound source becomes necessary—such as in a multi-talker conversation. Since humans can only attend to one auditory object at a time, auditory selective attention (ASA) switches between such competing sound sources are necessary^2^. These attention switches can happen involuntarily, e.g., when someone’s own name is called^3^ or by attention capturing noise such as a siren. Another scenario involves voluntarily attention switches, e.g., by actively focusing on a new speaker or location^4^. The presented study aims at investigating these voluntary attention switches under complex noise scenarios.

To investigate voluntary switching of ASA and assess the associated switch costs, Koch et al.^5^ developed a paradigm using dichotic playback. In this task, participants were presented with digit words ranging from one to nine (excluding five) spoken by a female and a male speaker. The digits were presented simultaneously by a target and distracting speaker in one ear each. The task was to determine, wether the target presented a digit below or above five. Between trials, the target gender could be changed. The reorientation to a different gender required an attention switch and introduced related switch costs in terms of worse task performance. Further, the congruence of stimulus content was varied, i.e., either target and distractor played congruent digits (both below or above five), or they played incongruent stimuli (e.g., target digit below five and distractor digit above five). Task performance was generally better for congruent trials. The interaction of congruence and attention switching supported the hypothesis that also the unattended distractor stimulus was processed.

While this research added to the understanding of voluntary switching between concurrent talkers, the dichotic paradigm does not reflect daily acoustic challenges. Although efforts to enhance the plausibility of studies by replicating realistic scenarios in laboratory settings are commendable, we still lack a comprehensive understanding of the acoustic and visual influences that shape real-life conversations in a cocktail party environment. A valuable overview of the challenges associated with striving for ecological validity can be found in Keidser et al.^6^.

Oberem et al.^7^ took a first step towards combining the number categorization task by Koch et al.^5^ with a more plausible acoustic scenario by using a binaural reproduction to create a lifelike acoustic environment for participants. In this approach, the target and distractor speakers were now played from one of eight spatial positions, which were equally distributed every $45^{\circ }$ on the horizontal plane. This allows for the investigation of the spatial relation between target and distractor. An illustration of the spatial distribution, as applied in the present study, is given in Fig. 3. While Oberem et al.^7^ could reproduce the switch costs and congruence effect by Koch et al.^5^, they also found interaction effects with the spatial configuration of target and distractor. This was also confirmed in further studies^8–10^. Their results showed higher switch costs for acoustically more challenging situations, e.g., when target and distractor were placed in the front and back of the listener as opposed to the left and right of the listener. An overview on this series of experiments on adults is given in Fels et al.^11^ However, real-life situations pose even greater acoustic challenges especially for children and older adults^9,12–14^. In adapting the binaural number categorization task by Oberem et al.^7^, Loh et al.^9^ investigated the impact of spatially distributed speech-shaped noise with a frequency spectrum similar to that of young children’s voices. The study further investigated the age effect by testing adults and children. No difference in attention switching was found between the children and young adults. However, children were more impaired by the noise than adults in terms of higher error rates and slower reaction times under noise. The results further suggested that children benefit less from the spatial separation of target and distractor than adults. This indicates that auditory spatial processing in relation to auditory selective attention develops over time.

While the work by Loh et al.^9^ took an important step towards understanding the effect of noise on ASA, they didn’t reflect real-world noise situations. However, studies directly investigating the effects caused by complex dynamic noise scenes including attention-capturing elements such as intelligible speech are sparse. One study highlighting how selectively listening to a conversation can reduce the perception of unattended stimuli is the auditory gorilla experiment presented by Dalton and Fraenkel^15^. Here, participants were asked to follow a conversation while ignoring a binaurally presented dynamic background scene. Most participants did not notice a distracting speaker who walked through the dynamic auditory scene while uttering the sentence “I am a gorilla.”. This finding suggests that ASA can suppress semantic content. However, the semantic information may influence cognitive load, as a study by Yadav et al.^16^ suggests. They conducted an auditory verbal serial recall task, investigating the impact of monaurally and binaurally presented noise including unintelligible speech and meaningful speech. The results showed that only the noise containing intelligible speech impaired performance. In an effort to better understand the influence of noise type on speech perception, Szalárdy et al.^17^ aimed to disentangle the effects of energetic (i.e., physical) and informational (i.e., behavioral) masking in multitalker situations. To do so, they presented participants with two concurrent speech streams. The loudness of the distracting stream was manipulated to be either slightly or substantially louder or softer than the target stream (± 5 dB and 10 dB). Since concurrent speech was used, all distractors induced informational masking, while louder distractors also induced energetic masking. Although task performance was also impaired by the moderately louder distractor indicating energetic masking, the results showed that the condition in which the distracting stream was only 5 dB softer than the target was the most difficult for participants. This finding suggests that informational masking can strongly affect speech perception even when the target speech remains highly intelligible. It further underlines the importance of investigating meaningful distractors such as speech instead of simple noise types to consider effects caused by informational as well as energetic masking.

In addition to the noise type, the study by Yadav et al.^16^ further investigated the influence of monaural and binaural presentation of the noise sources. Contrary to the findings on ASAby Oberem et al.^7^, no effect of spatial separation in terms of diotic and binaural presentation of the noise was found for the serial recall. Using a similarly complex noise a study by Leist et al.^18^, investigated listening comprehension and word identification in a monaural and binaural classroom noise scenario. Here, the monaural noise conditions showed higher levels of impairment in a word identification task than the binaural conditions. The authors argue that the participants used spatial release from masking in the binaural condition to separate the target words from the noise sources. In line with this, Valzolgher et al.^19^ showed that source localization is also affected by the complexity of the noise source in a virtual reality (VR) based study. Additionally, participants reported higher listening effort in more complex scenes.

Although most of the mentioned studies did not assess ASA directly, they all focused on aspects highly relevant to cocktail party situations - such as localization, word recognition, or processing of semantic content. Given the complexity of processes involved in these scenarios, the cognitive effects introduced by complex background sounds remain unclear. For example, noise can increase listening effort, while task performance is unaffected^20,21^. However, the relationship between auditory attention and listening effort is not thoroughly researched. Therefore, in order to investigate the effects of noise type in conjunction with associated cognitive costs, listening effort was measured in terms of an extended version of the NASA-Task Load Index (NASA-TLX) questionnaire^22–24^.

While the referenced works improved the realism of the acoustic scenarios, efforts to create closer-to real-life scenarios are made by incorporating visual representations using VR environments (see, e.g., Rizzo and Koenig^25^, or Breuer et al.^10^). Though VR can enhance ASA and cognitive performance, which was shown by fewer errors and faster responses in studies by Wan et al.^26^ and Li et al.^27^, other findings suggest task performance in VR may decline due to perceptual overload (Makransky et al.^28^). Notably, Breuer et al.^10^ replicated the previously described categorization task^5,7^ in VR. They found the same overall effects of switch cost, spatial relation of target and distractor and congruence in the VR-based^10^ and the screen-based version by Loh et al.^9^. However, the task performance was improved in the VR experiment, supporting the idea that VR can increase task focus.

The underlying cause remains uncertain, but one explanation is that a stronger sense of presence enhances attention and performance. To assess presence, the Slater-Usoh-Steed (SUS) questionnaire^29^ is an established measure. According to this questionnaire, the sense of presence classifies the feeling of being in the virtual scene and to what extend the virtual scene resembles a real environment. Prior research suggests that matching audiovisual reproduction^30,31^ or binaural reproduction and room acoustic simulations^32^ can increase the perceived presence. Considering these results, the question arises whether the realistic classroom noise can also enhance the perception of presence.

While the presented study was conducted using adult participants, the overall goal of the research project is to investigate developmental effects in children, since it is known that children are more severely affected by noise than adults^9,18,33^ and their ability to selectively attend sound sources develops over age^9,34,35^. By testing adult participants, the present study establishes a foundation and a reference point for future studies investigating children. The proposed scenario is expected to be suitable also for young adults, since they experience situations similar to the one presented here during seminars or lectures and should thus be able to relate to the overall scenario.

The main objective of this study was to investigate the effect of complex noise scenarios on ASA. It builds upon the works by Oberem et al.^7^, Loh et al.^9^ and Breuer et al.^10^, who lay the foundation to investigate ASA in more realistic everyday situations. Therefore, a strongly simplified approach using white noise was compared to a more realistic non-stationary classroom noise with and without speech. The first variant of the new classroom noise was created following the noise scenes implemented by Yadav et al.^16^ and Leist et al.^18^ and includes a number of different typical classroom or office sounds, such as chairs and pages being turned. As a second complex noise condition, the classroom scene was supplemented by intelligible speech snippets.

The complex auditory scenes, with and without disruptive speech signals, are expected to draw more attention and impair task performance more than white noise. As a result, we expect higher attention switch costs, a stronger congruence effect, and reduced benefit of spatial separation between target and distractor in the complex noise conditions compared to silence and white noise. This decline in task performance highlights that non-stationary, realistic noise is more distracting than simplified noise scenarios. To better understand how these acoustic conditions affect perception, we also measured listening effort and presence in the virtual environment using adapted versions of the NASA-TLX and SUS questionnaires. In line with task performance, listening effort is expected to increase under complex classroom noise, while a higher sense of presence is anticipated in these acoustic scenes, reflecting greater audiovisual plausibility. Together, the results will emphasize the need for more plausible noise scenarios to improve real-world predictions, for instance in hearing aid research.

## Methods

### Participants

A group size of 24 participants (age: 20–38 years, M = 24.13 years, SD = 3.56 years, 6 female, 18 male) was chosen based on previous studies using the paradigm^5,7,9,10,36^. All participants were required to have fluent German skills. All but one of them were native German speakers. Further requirements were normal hearing (within 25 dB [HL]^37^) and (corrected to) normal vision. The hearing was measured by an Auritec Ear 3.0 audiometer using an ascending pure tone audiometry between 250 Hz and 8kHz^38^. Further, normal or corrected to normal vision acuity (20/30) was measured by a Snellen chart^39^. Color vision was tested using a subset of Ishihara color charts (charts: 1, 2, 4, 8, 10, 14, according to instructions for quick testing)^40^. None of the participants participated in a listening experiment on ASA within the last six months before the study. A statement of non-objection was obtained from the Medical Ethics Committee at RWTH Aachen University with the protocol number EK 395-19. Informed consent was obtained form all participants before the participant screening and the study. The study was performed in accordance with the Declaration of Helsinki^41^. All participants received an allowance of 10 €.

### Experimental task and design

**Fig. 1:**
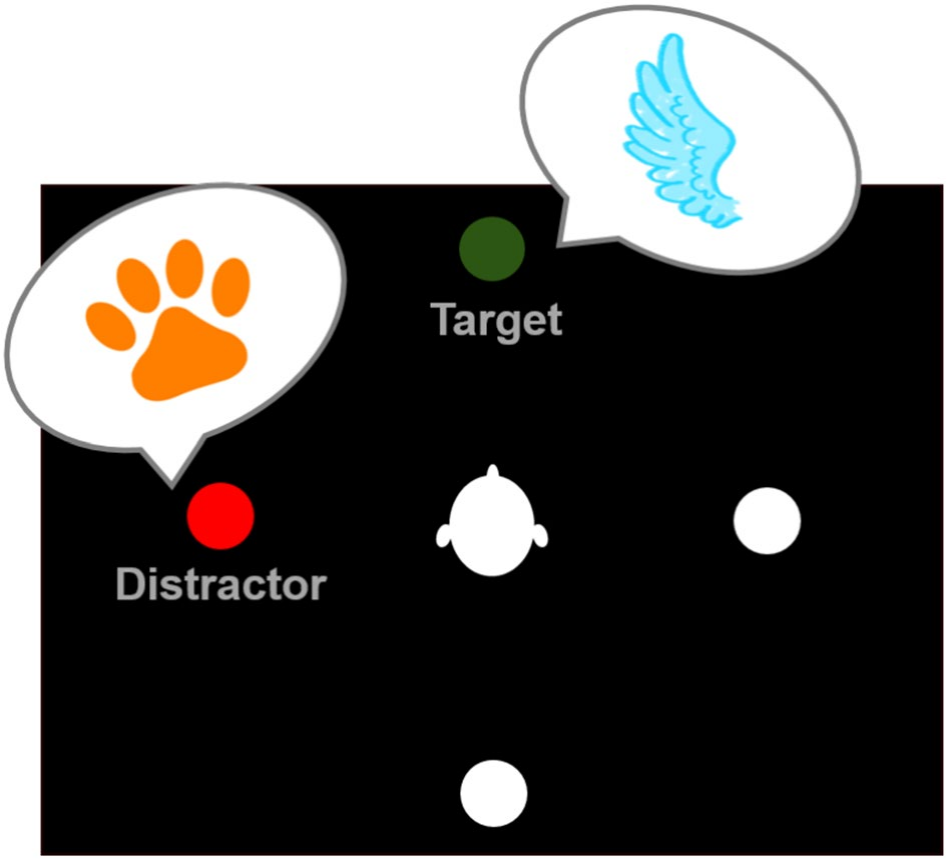
Experimental design.

Based on previous studies by Koch et al.^5^ and Oberem et al.^7^, the present study used a spatial classification task to measure voluntary switching of ASA. Given the overall project goal, a child-appropriate version proposed by Loh et al.^9^ and validated in a virtual classroom environment by Breuer et al.^10^ was used. In this task, participants are immersed in a virtual classroom environment and seated in the middle of a circle of chairs. A schematic of the experimental task is illustrated in Fig. 1. Participants are asked to direct their auditory attention to one of four possible positions (front, back, left, right) and classify whether the animal name played from this position belonged to a flying animal or not. The respective target position was cued at the beginning of each trial using the sound of snapping fingers played back from the target position. Additionally to the target stimulus, a distractor was played at the same time, but from a different position.

During the experiment, the spatial and temporal representation, as well as the content of the auditory stimuli were manipulated each trial. Additionally, three noise types were introduced and tested against a silent baseline. These independent variables are described in detail in the following section. As a measure of task performance, the reaction times (RTs) in ms and error rates (ERs) in % were recorded.

#### Attention transition

**Fig. 2:**
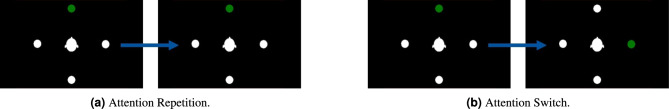
Illustration of the variable attention transition with the levels *repetition* and *switch*.

At the beginning of each trial, one of four possible positions was marked as the target position. This position could either be repeated (e.g., *front-front*, Fig. 2a) or switched (e.g., *front-right*, Fig. 2b) between trials. Since the switching of the target position requires a refocusing of the auditory attention, an attention transition (AT) is introduced. Contrary to the target position, the distractor position was changed for each trial.

#### Congruence

During each trial, the auditory target and distractor stimuli could either both belong to the same category, or to different ones. This is described as the auditory congruence (C). If both stimuli played a flying animal name (e.g., *bee–dove*), they were congruent. If the target played a flying animal name while the distractor played a non-flying animal name, or vice versa (e.g., *bee–cat*), the stimuli were considered incongruent. The incongruent scenario is illustrated in Fig. 1.

#### Target-Distractor Position-Combination

**Fig. 3:**
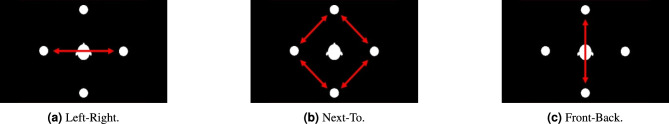
Illustration of the variable target-distractor position-combination with the levels *left-right*, *next-to*, and *front-back*.

The combination of target and distractor position was varied between trials since the distractor position was changed each trial. The target-distractor position-combination (TD) described the spatial relation between the target and distractor stimuli. It had three levels and could either be left-right (Fig. 3a), next-to (e.g., *left-front*, Fig. 3b), or, front-back (Fig. 3c).

#### Noise

**Fig. 4:**
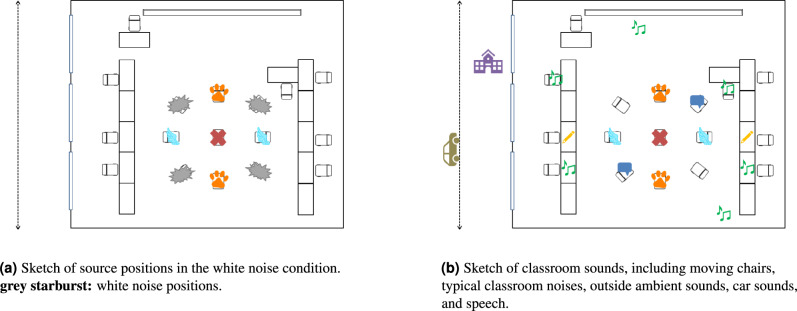
Sketch of noise types distributed within the virtual classroom scenario. Red cross: participant position, wing and paw: target positions.

The noise (N) conditions silence, white noise (Fig. 4a), classroom noise without (classroom) and with intelligible speech (speech, Fig. 4b) were presented in blocks and balanced across the participants according to a 4 × 4 latin square. The playback started with the first cue sound and stopped after the feedback of the last trial.

### Questionnaires

#### Listening effort

To assess subjective listening effort the NASA-TLX questionnaire was used^21,22^. It includes six dimensions: Mental Demand, Physical Demand, Temporal Demand, Performance, Effort and Frustration. Since not all of these measures are applicable for listening experiments, Bologna et al.^23^ suggest an adapted version using the domains Mental Demand, Perceived Effort, Task Difficulty, Frustration, and Performance. These dimensions typically have a negative connotation. However, according to a review on measuring listening effort by Francis and Love^24^, effortful situations do not need to be perceived negatively. They give the example of running a marathon, which is effortful, but can be perceived positively as an accomplishment. Therefore, they argue to include the question of well-being as an additional dimension, i.e., how participants felt during the task. All questions were answered on a scale from 1 (very low) to 7 (very high). Both the German and English versions of the questions are stated in the supplementary material online.

#### Presence

The motivation to use a virtual environment is to increase the plausibility of the experiment and thus obtain participant responses that resemble real-world behavior. To achieve this, participants need to experience a sense of presence, i.e., they feel as though they are inside the virtual environment^42,43^. To assess the sense of presence, a German version of the SUS presence questionnaire^29^ was used. It consists of six questions asking about the sense of being in the virtual scene, whether the participants forgot about the real world, and to what extent the virtual scene is perceived as a real place. All questions were rated on a 7-point scale with question-specific end points and are provided in the supplementary material online.

### Stimulus material

In accordance with a previous study by Loh et al.^9^, audio recordings of eight animal names in the German language were used. The names could be categorized into flying animals (“Biene” (bee), “Ente” (duck), “Eule” (owl), “Taube” (dove)), and non-flying animals (“Schlange” (snake), “Ratte” (rat), “Robbe” (seal), “Katze” (cat)). The stimuli were recorded in the anechoic chamber of the Institute for Hearing Technology and Acoustics with a female adult (26 years) and a male child (5 years) using a Neumann TLM 170 condenser microphone and a Zoom H6 recorder at a sampling rate of 44.1 kHz. The stimuli and respective documentation are available in the ChildASA dataset by Loh and Fels^44^. To have equally long stimuli, the “change tempo” algorithm provided by the software Audacity^45^ was used to adjust the stimuli length to 600 ms. The female adult and male child speakers were chosen by Loh et al.^9^ to reflect daily listening situations in German elementary schools, where many teachers are female. The measured fundamental frequencies of the male child and female adult are similar (209.6 Hz and 240.2 Hz). The similarity of both voices is expected to have less influence on task difficulty in this paradigm than the other factors such as spatial distribution of target and distractor. This was shown by a previous study by Lawo et al.^46^ who used a dichotic version of the paradigm with male and female voices. When investigating the impact of switching the target gender vs. the spatial selection, they found that the auditory switch costs are higher, when the participants had to reorient their spatial attention compared to switching the gender.

The acoustic cue was the sound of snapping fingers obtained from Freesound^47^ and was adjusted to a total cue duration of 450 ms.

### Noise design

To investigate the impact of more realistic, non-stationary sounds compared to classical approaches such as white noise, three different noise types were designed: spatially distributed white noise, spatially and temporally distributed classroom sounds with and without intelligible speech.

#### White noise

The white noise condition was designed using four EBU-R128 loudness-normalized sources playing a white noise sound from Freesound^48^. Based on previous studies, they were placed on the horizontal plane at angles of 45$^\circ$, 135$^\circ$, 225$^\circ$, and 315$^\circ$, at a distance of two meters from the participant (see Fig. 4a)^9^.

#### Classroom noise

Typical inside and outside classroom sounds were used to create a realistic and dynamic noise environment as illustrated in Fig. 4b. Two static noise sources were placed at 90$^\circ$ to the left and right of the participant (Fig. 4bpencil symbol). These sources emitted various sounds of pens being scratched, paper being cut, pages being turned in a ring binder, and pens being searched for in a pencil case. Another source randomly played several short sounds of chairs being moved from a predefined set of positions (Fig. 4bnote symbol). All inside sounds were recorded in an anechoic chamber using a Neumann TLM 170 condenser microphone and a Zoom HD6 recorder. A fourth sound source was placed outside of the classroom to simulate a busy schoolyard (Fig. 4bhouse symbol). This source consisted of a recording of a German playground from Freesound^49^. Finally, an artificial car sound, taken from the IHTA park model^50^, moved past the classroom, simulating cars driving by (Fig. 4bcar symbol). All sounds were looped with different lengths to create a sustainable background noise that is never repeated.

#### Classroom noise with speech

To account for background chatter by classmates, speech was added to the realistic classroom noise condition. Two sound sources were placed at 45$^\circ$ front-right and back-left in the same distance as the target sounds (see Fig. 4bspeech bubble symbols). These sources emitted whispers and calls from other pupils such as “Wie lange dauert das noch?” (How much longer will this take?) or “Verstehst du das?” (Do you understand that?). The spoken phrases consisted of individual sentences, some of which were answers to others. Due to the random playback, pauses of up to 36 seconds without speech could exist between the speech signals. The whispered statements were recorded with two male and one female student in the VR-LAB of the Institute for Hearing Technology and Acoustics of RWTH Aachen University using a Rode NT1-A microphone and the software REAPER. Further speech signals by a female student in normal voice were recorded in the same way as the inside classroom sounds described above.

### Audiovisual reproduction

#### Acoustic reproduction

The auditory stimuli were produced using the Virtual Acoustics (VA)auralization framework^51^ version 2021 and the respective Unity package^52^. The dynamic binaural synthesis of the direct sound used a generic c head-related transfer function (HRTF) of the IHTA artificial head with a resolution of $5^\circ \times 5^\circ$^53^. This allowed for spatial placement of the stimuli and took the tracked head movements of the participants into account. All stimuli were played back with a static reverberation of 0.19 s in low frequencies and 0.76 s in higher frequencies as defined by the BinauralArtificialReverb renderer provided in VA^51^. Sennheiser HD 650 headphones were used for the auditory playback. Headphone equalization filters^54^ were measured for each participant using the ITAtoolbox for Matlab^55^ to account for individual differences. All stimuli and noise conditions were leveled to an overall level of 60 dB(A) based on silent working scenarios in real classroom situations^56–59^, aiming at a signal-to-noise ratio (SNR) of 0 dB(A). For the realistic classroom noise scenarios, the level was measured and averaged over one minute.

#### Visual reproduction

**Fig. 5:**
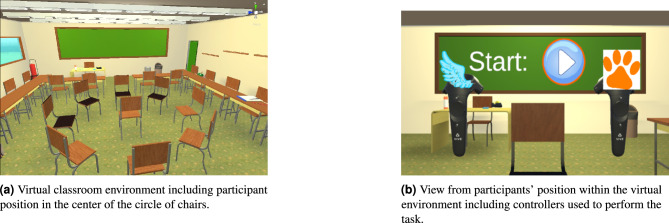
Virtual environment with controllers.

**Fig. 6:**
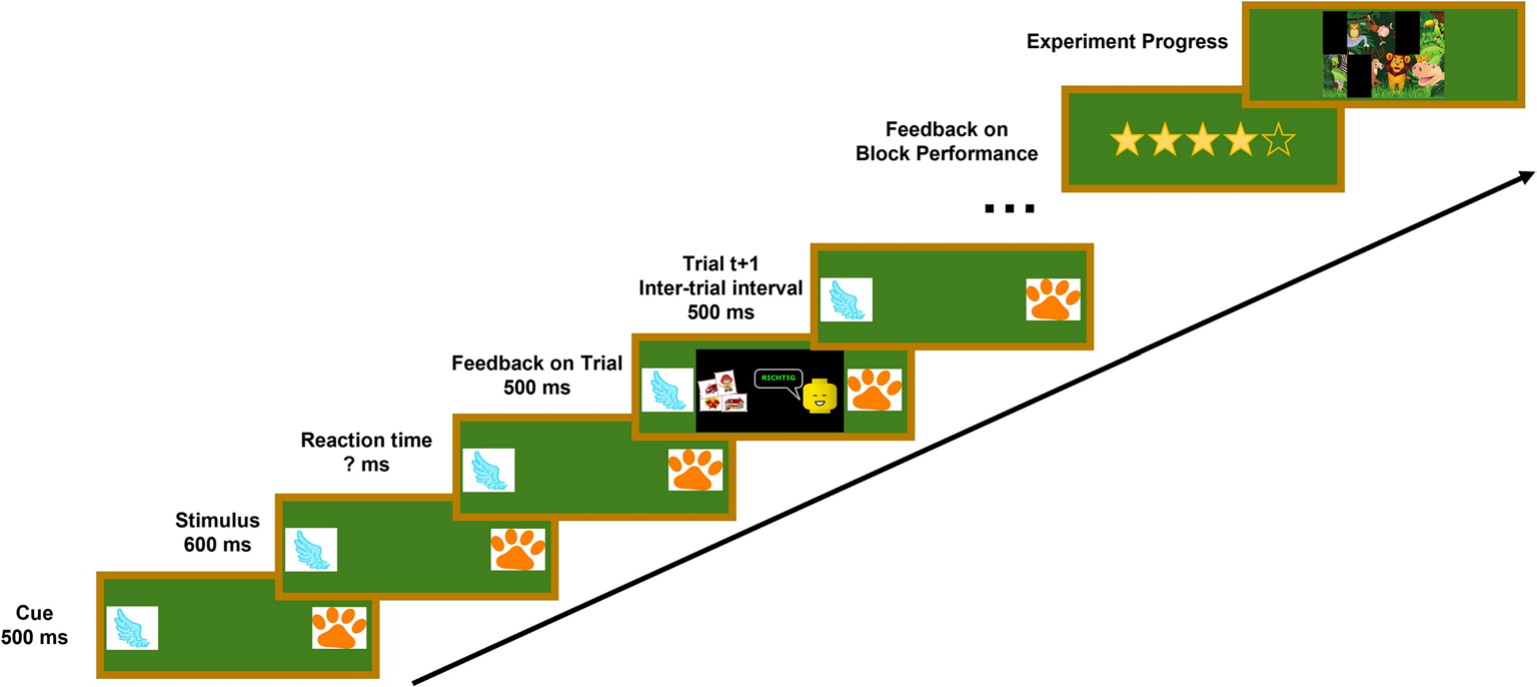
Illustration of structure of one trial within the experiment, as well as feedback and progress indication after each block.

For visual reproduction, the virtual classroom model introduced by Breuer et al.^10^ was used (see Fig. 5a). The model was created using SketchUp make 2016^60^ and adapted in Unity 2019.4.21f^61^ including furniture, outdoor environment, and lighting. The dimensions of the virtual classroom were chosen based on the requirement to fit a circle of chairs with a diameter of 4 m, which represented the stimulus positions. The final classroom had the measurements of $l \times w \times h = 10 \times 9 \times 3 \, \text {m}^3$. During the experiment, all instructions and feedback were displayed on a large blackboard in front of the participants (see Figs. 5 and 6).

The Unity project including the VA version, Matlab code and ITAtoolbox used for this study are available on Zenodo^62^.

The virtual environment was presented using an HTC Vive Pro Eye head-mounted display (HMD). Before the experiment, the participants were instructed to adjust the HMD to fit comfortably. The participants could interact with the scene using the HTC Vive controllers (see Fig. 5b). To visualize which controller belonged to which response category, images of a wing and a paw were attached to the virtual controller models. A frame rate of 90 fps was achieved during the experiment to ensure an adequate visual reproduction.

### Experimental procedure

Figure 6 visualizes the structure of each trial. Each trial began with the auditory cue and the cue-stimulus interval of 500 ms. Afterward, the auditory stimuli were played back and the participants had unlimited time to respond. The reaction times measurement began directly after the stimulus playback and continued until participants provided a response. Feedback on the correctness of the answer was displayed for 500 ms after each response. This was followed by an inter-trial interval of 500 ms. To measure all time intervals, the *Stopwatch* class from the C# standard library was used, which is included in the Unity game engine scripts controlling the experiment. The Unity project including all stimuli and scripts is available on Zenodo^62^.

At the start of the session, a training phase with 32 trials of increasing difficulty was introduced to familiarize the participants with the task and the noise. During the training, participants were instructed to answer as quickly as possible. To increase motivation, participants received a feedback on whether the answer was correct directly after each trial. The experiment was divided into four blocks of 180 trials each. Attention transition, congruence and target-distractor position-combination were varied between trials. Noise was manipulated between blocks, i.e., the different noise types were presented in different experiment blocks. After 90 trials, an optional break was introduced.

To keep the participants motivated and as a gamification element, the number of correct answers was visualized by three to five golden stars displayed on a blackboard in front of the participant as intermediate feedback after 90 trials (see Fig. 6). At the end of a block, another feedback on the overall performance during the block was given by showing the respective amount of stars. Further, the progress of the experiment was illustrated by revealing parts of an image. After each block, one part of the image, which was also displayed on the blackboard, was revealed. Thus, in the beginning only a black rectangle was displayed and at the end of the experiment, the whole image was visible.

A mandatory break was taken after each block of 180 trials (i.e., after each noise condition). Participants had to take off the HMD and leave the virtual environment. They filled out the questionnaires regarding listening effort and their experience in the virtual environment on a computer screen.

The whole experiment including participant screening and questionnaires lasted about 90 minutes.

The experiment was carried out at the Institute for Hearing Technology and Acoustics, RWTH Aachen University, Germany, in an acoustically treated hearing booth ($l \times w \times h = 2.3 \times 2.3 \times 1.98 \, \text {m}^3$), which offered a quiet environment.

### Hypotheses

Attention Transition: Since the repositioning of the target stimulus requires a transition of auditory attention, switch costs in terms of longer RTs and increased ERs are expected.

Congruence: Given the content of the target and distractor stimuli, better task performance is expected in congruent than incongruent trials, which would be reflected in lower ERs and shorter RTs.

Target-Distractor Position-Combination: To present the spatially distributed stimuli, a generic binaural reproduction was used, which can cause front-left confusions as well as in-head localization of the stimuli. It is thus hypothesized that the left-right target-distractor position combination yields the best task performance in terms of fastest RTs and lowest ERs. Next-to conditions are expected to result in worse performance. Front-back conditions are expected to yield the worst performance (slowest RTs and highest ERs).

Noise: Regarding the noise types, task performance is expected to decrease with increasing noise complexity. The non-stationary classroom sounds are expected to capture attention, while speech also introduces informational masking. Thus, best performance is expected for the silent baseline and white noise conditions (fastest RTs and lowest ERs). Task performance is expected to be impaired for the classroom noise and worst for the classroom noise with speech.

The noise is expected to interact with the factors stated above in a way that the effects are expected to be increased by the noise.

Interaction of Noise and Attention Transition: The classroom and speech noise are expected to increase switch costs, which will be reflected in longer RTs and higher ERs in switch compared to repetition trials under complex noise. In case of a weak effect of noise type, an interaction will only be visible in attention switches.

Interaction of Noise and Congruence: The classroom and speech noise are expected to increase the congruence effect, which will be reflected in longer RTs and higher ERs in incongruent than congruent trials under complex noise. In case of a weak effect of noise type, an interaction will only be visible in incongruent trials.

Interaction of Noise and Target-Distractor Position-Combination: The classroom and speech noise are expected to increase the effect of spatial separation of sound sources. Given the large separation of target and distractor in left-right and next-to trials, a decrease in task performance in terms of longer RTs and higher ERs is expected only for the front-back condition.

Listening Effort: Perceived listening effort is expected to be higher for the classroom and speech noise than for silence and white noise, since the complex noise types are expected to be harder to filter than stationary noise. This will be represented by higher scores in the adapted NASA-TLX questionnaire.

Presence: Since the sense of presence is related to the plausibility of a virtual scene, the presence is expected to be lowest without any noise. Adding classroom noise will increase the sense of presence and it will be highest for the noise including speech. This will be reflected in higher scores in the SUS questionnaire.

## Results

In each trial, reaction times in ms and error rates in % were evaluated. To process the raw data, all training trials, the first trial of each block, and trials following a false response were filtered. Outliers were treated by removing all trials with RTs lower than 50 ms and higher than 6000 ms. Further, a Z-transformation of the RTs was calculated per participant, and trials exceeding ± 2 z were removed. A total of 4 % of trials were removed as outliers. In the RTs analysis, the error trials were removed additionally. Four independent variables noise (silence vs. white noise vs. classroom noise vs. classroom with speech), attention transition (repetition vs. switch), congruence (congruent vs. incongruent) and target-distractor position-combination (left-right vs. next-to vs. front-back) were investigated. Repeated measures analyses of variances (ANOVAs)(N $\times$ AT $\times$ C $\times$ TD) were performed for the RTs and ERs separately. Using a repeated measures ANOVA has proven robust for the data at hand, even if the assumptions of normal distribution were violated^63,64^. Given the high number of variables that results from the experimental task, the experimental design is very complex. Thus, in the following, only significant results and relevant interactions with the variable noise are discussed. Main effects that are part of an interaction are not described separately. An overview on all results and interactions is displayed in Table 1.

**Table 1:** ANOVA (N $\times$ AT $\times$ C $\times$ TD) results for reaction time and error rate.

Within-Group Variable	Reaction time				Error rate			
	*df*	*F*	*p*	$\eta ^{2}_{p}$	*df*	*F*	*p*	$\eta ^{2}_{p}$
AT	**(1,22)**	**6.115**	**0.022**	**0.217**	(1,23)	2.483	0.129	0.097
C	**(1,22)**	**5.688**	**0.026**	**0.205**	**(1,23)**	**332.743**	< **0.001**	**0.935**
TD	**(1.235,44)**$^{a}$	**19.533**	< **0.001**	**0.470**	**(1.482,46)**$^{a}$	**167.018**	< **0.001**	**0.879**
AT$\times$C	(1,22)	0.366	0.551	0.016	(1,23)	1.987	0.172	0.080
AT$\times$TD	**(2,44)**	**6.598**	**0.003**	**0.232**	(1.236,46)$^{a}$	0.209	0.703	0.009
C$\times$TD	**(1.483,44)**$^{a}$	**6.337**	**0.009**	**0.224**	**(1.329,46)**$^{a}$	**109.948**	< **0.001**	**0.827**
AT$\times$C$\times$TD	(2,44)	0.755	0.476	0.033	(2,46)	0.732	0.486	0.031
N	(3,66)	0.660	0.580	0.029	**(3,69)**	**5.910**	**0.001**	**0.204**
N$\times$AT	(3,66)	2.284	0.087	0.094	(3,69)	2.363	0.079	0.093
N$\times$C	**(3,66)**	**3.179**	**0.030**	**0.126**	**(3,69)**	**4.511**	**0.006**	**0.164**
N$\times$TD	(6,132)	1.057	0.392	0.046	(6,138)	1.353	0.238	0.056
N$\times$AT$\times$C	(3,66)	0.690	0.561	0.030	(3,69)	1.994	0.123	0.080
N$\times$AT$\times$TD	(6,132)	0.196	0.977	0.009	(4.075,138)$^{a}$	1.579	0.185	0.064
N$\times$C$\times$TD	(6,132)	0.604	0.727	0.027	(6,138)	1.123	0.352	0.047
N$\times$AT$\times$C$\times$TD	(3.815,132)$^{a}$	0.485	0.738	0.022	(6,138)	1.459	0.197	0.060

N = noise; AT = attention switch; C = congruence; TD = target-distractor position. $^{a}$ Greenhouse-Geisser correction applied due to significant in the sphericity test. All significant main and interaction effects are highlighted.

### Reaction times

For the evaluation of RTs one participant was eliminated due to a too small number of valid repetitions.

The ANOVA revealed a main effect of AT, $F(1,22) = 6.115, p = 0.022, \eta ^{2}_{p} = 0.217$, indicating lower RTs in repetition than in switch trials (1081.2 ms vs. 1098.9 ms).

The significant main effect of C, $F(1,22) = 5.688, p = 0.026, \eta ^{2}_{p} = 0.205$, indicates lower RTs for congruent than incongruent trials (1070.3 ms vs. 1109.9 ms). Mauchly’s test indicated that the assumption of sphericity were violated for TD ($\chi ^{2} = 20.281, p < 0.001$). Degrees of freedom were corrected using Greenhouse-Geisser estimates $\epsilon _{gg} = 0.618$.

The main effect of TD was significant, $F(1.235,44) = 19.533, p < 0.001, \eta ^{2}_{p} = 0.879$. Bonferroni-adjusted post-hoc test revealed significantly lower RTs for the left-right (995.6 ms) compared to the next-to (1093.6 ms), and the front-back (1181.0 ms) conditions ($p < 0.001$). The comparison of next-to and front-back was also significant ($p = 0.027$).

The interaction of AT $\times$ TD was significant, $F(2,44) = 6.598, p = 0.003, \eta ^{2}_{p} = 0.232$. Bonferroni-adjusted post-hoc tests showed lower RTs in repetition compared to switch trials for front-back condition (1156.8 ms vs. 1205.1 ms, $p < 0.001$).

For the interaction of C $\times$ TD, Mauchly’s test indicated that the assumption of sphericity was violated ($\chi ^{2} = 9.006, p = 0.011$). Therefore, the degrees of freedom were corrected using Greenhouse-Geisser estimates ($\epsilon _{gg} = 0.618$). The interaction was significant, $F(1.483,44) = 6.337, p = 0.009, \eta ^{2}_{p} = 0.224$. Bonferroni-adjusted post-hoc tests showed lower RTs in congruent than incongruent trials for front-back condition (1128.6 ms vs. 1233.3 ms, $p = 0.010$).

The interaction of N $\times$ AT was not significant, $F(3,66) = 2.284, p = 0.087, \eta ^{2}_{p} = 0.094$. Bonferroni-adjusted post-hoc tests indicated a tendency towards higher RTs in switch than repetition trials in the white noise condition (1104.734 ms vs. 1056.320 ms $p = 0.004$).

**Fig. 7:**
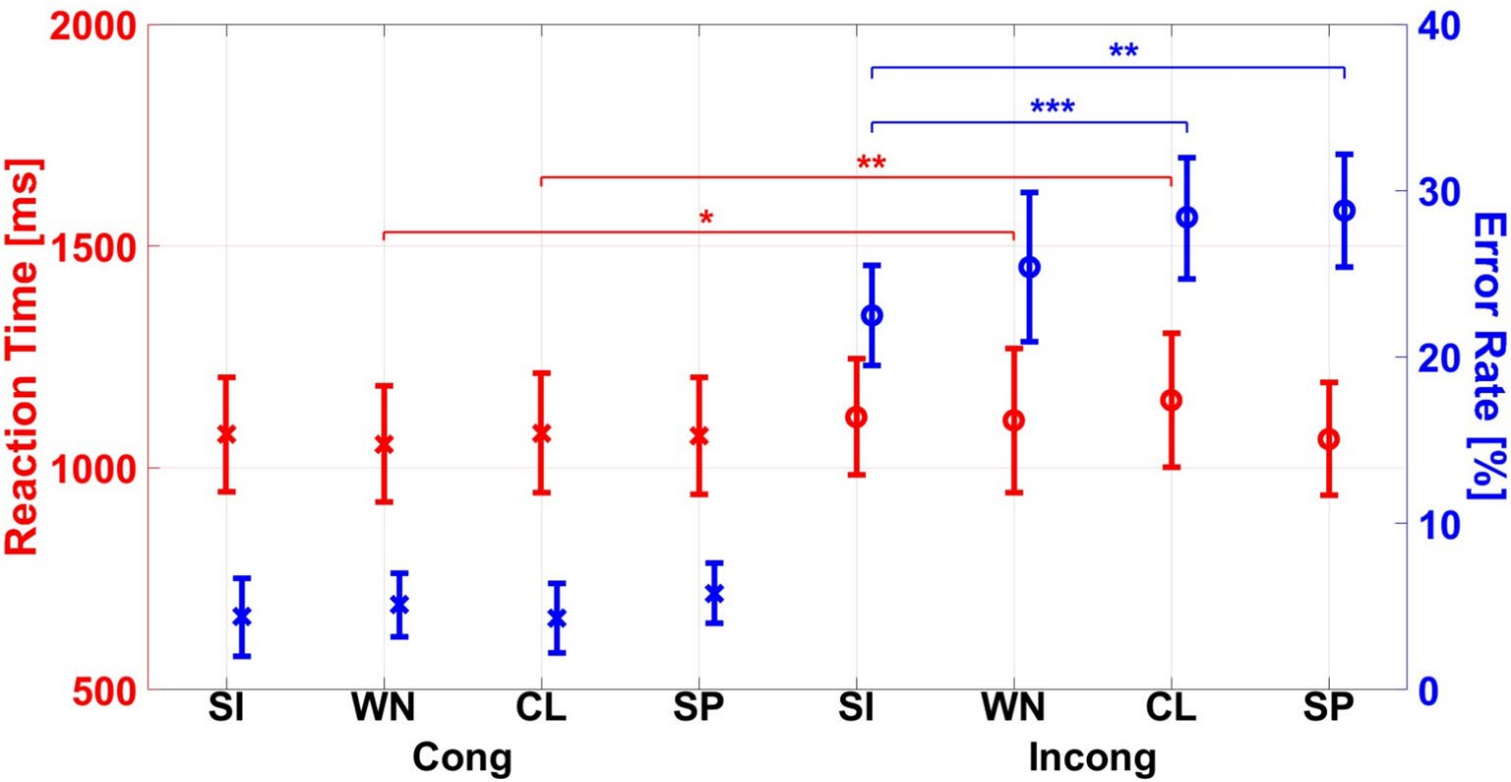
Interaction effects by noise (silence (SI), white noise (WN), classroom (CL), speech (SP) and congruence on error rate (blue) and reaction time (red). Mean values are accompanied by the 95 % confidence intervals. Significance levels are indicated by asterisks, where * = *p* < 0.05, ** = *p* < 0.01, *** = *p* < 0.001.

The interaction of N $\times$ C was significant, $F(3,66) = 3.179, p = 0.030, \eta ^{2}_{p} = 0.126$ (see Fig. 7), indicating lower RTs for congruent than for incongruent trials under white noise (1053.8 ms vs. 1107.2 ms $p = 0.038$), and classroom noise (1078.7 ms vs. 1152.6 ms, $p = 0.003$).

### Error rates

The main effect of C was significant, $F(1,23) = 332.743, p < 0.001, \eta ^{2}_{p} = 0.935$, indicating lower ERs for congruent than incongruent trials (4.9 % vs. 26.3 %).

Mauchly’s test indicated that the assumption of sphericity is violated for TD ($\chi ^{2} = 9.472, p = 0.009$). Greenhouse-Geisser estimates $\epsilon _{gg} = 0.741$ were used to correct the degrees of freedom. The main effect was significant, $F(1.482,46) = 167.018, p < 0.001, \eta ^{2}_{p} = 0.879$. Bonferroni-adjusted post-hoc tests showed significantly lower ERs for left-right (7.6 %) compared to next-to (12.0 %) and front-back (27.1 %), as well as significantly lower ERs for next-to than front-back ($p < 0.001$). The main effect of AT was not significant.

For the interaction of C $\times$ TD Mauchly’s test indicated that the assumption of sphericity is violated ($\chi ^{2} = 15.448, p < 0.001$). Therefore, the degrees of freedom were corrected using Greenhouse-Geisser estimates ($\epsilon _{gg} = 0.618$). The interaction of C $\times$ TD was significant, $F(1.329,46) = 109.948, p < 0.001, \eta ^{2}_{p} = 0.827$. Bonferroni-adjusted post-hoc tests revealed significantly lower ERs for all TD combinations (left-right, next-to, front-back) in congruent than incongruent trials (3.9 %, 4.6 %, 6.3 % vs. 11.3 %, 19.5 %, 47.9 %, all $p < 0.001$).

The ANOVA revealed a main effect of N, $F(3,69) = 5.910, p = 0.001, \eta ^{2}_{p} = 0.204$. Bonferroni-adjusted post-hoc tests showed significantly higher ERs for classroom noise compared to silence (16.3 % vs. 13.4 %, $p = 0.003$). The comparison of speech (17.3 %) and silence was also significant ($p = 0.017$).

The interaction of N $\times$ AT was not significant, $F(3,69) = 2.363, p = 0.079, \eta ^{2}_{p} = 0.093$. Bonferroni-adjusted post-hoc tests indicate a tendency towards higher ERs in switch than repetition trials in the classroom noise with speech (18.9 % vs. 15.7 %, $p = 0.011$). There was also a tendency towards lower ERs in the silence condition than the speech noise condition (13.9 % vs. 18.9 %, $p < 0.001$) in switch trials, as well as a tendency towards lower ERs in the silence than the classroom noise (13.0 % vs. 16.4 %, $p = 0.012$) in repetition trials.

The interaction of N $\times$ C was significant, $F(3,69) = 4.511, p = 0.006, \eta ^{2}_{p} = 0.164$, see Fig. 7. Bonferroni-adjusted post-hoc tests indicated lower ERs for congruent than in incongruent trials for all noise conditions (silence 4.4 % vs. 22.5 %, white noise 5.1 % vs. 25.4 %, classroom 4.3 % vs. 28.4 %, speech 5.8 % vs. 28.8%, all $p < 0.001$). Further, the silent condition yielded significantly less errors than the classroom noise (22.5 % vs. 28.4 %, $p = 0.001$) and the speech noise (28.8 %, $p = 0.01$) in incongruent trials (see Fig. 7).

**Fig. 8:**
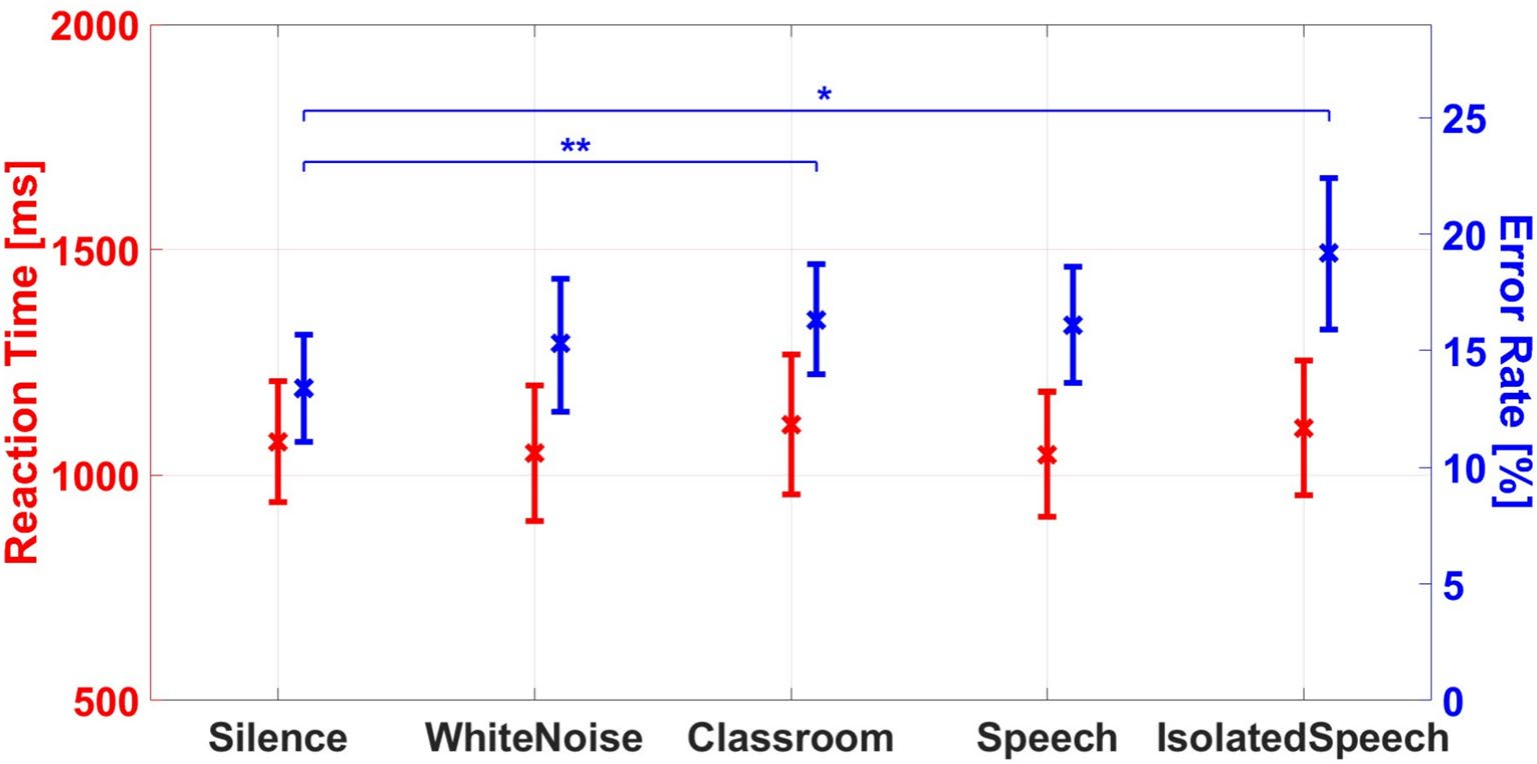
Main effects of the noise typed with additional isolated speech trials ($N_{sp}$) on error rate (blue) and reaction time (red). Mean values are accompanied by the 95 % confidence intervals. Significance levels are indicated by asterisks, where * = *p* < 0.05, ** = *p* < 0.01, *** = *p* < 0.001.

### Isolated speech trials

During the classroom noise including speech, the speech samples were played randomly during the respective experiment block. Thus, pauses of up to 36 seconds without speech occurred, which is long compared to a trial length of around 3–4 seconds. Thus, not every trial in the noise with speech condition actually contained disturbing speech files. To get further insight into the direct impact of speech on the ASA, all trials during which speech was played were filtered and grouped into a subset of the previous noise condition. Here all trials were selected during which speech was present at any time starting from the auditory cue. A second analysis was performed with this new noise condition of isolated speech trials. The raw data was again preprocessed and treated for outliers as described above. Subsequently, exploratory one-way ANOVAs were conducted with the resulting five noise conditions ($N_{sp}$) for RTs and ERs.

For the evaluation of RTs four participants were eliminated due to a too small number of valid trials. For $N_{sp}$ Mauchly’s test indicated that the assumption of sphericity was violated ($\chi ^{2} = 35.353, p < 0.001$). Greenhouse-Geisser estimates $\epsilon _{gg} = 0.555$ were used to correct the degrees of freedom. Similar to the previous analyses, the main effect of the new noise conditions on RTs was not significant, $F(2.222,76) = 1.300, p = 0.285, \eta ^{2}_{p} = 0.064$, (see Fig. 8).

Mauchly’s test also indicated that the assumption of sphericity was violated for the ERs ($\chi ^{2} = 30.937, p < 0.001$). Therefore, the degrees of freedom were corrected using Greenhouse-Geisser estimates ($\epsilon _{gg} = 0.583$). The main effect of the new noise conditions was significant, $F(2.330,76) = 5.391, p = 0.005, \eta ^{2}_{p} = 0.190$. Bonferroni-adjusted post-hoc tests showed significantly lower ERs for silence compared to classroom noise (13.4 % vs. 16.1 %, $p = 0.005$), as well as trials with isolated speech (19.2 %, $p = 0.038$). None of the other comparison of noise conditions were significant ($p > 0.05$). Thus, the results are comparable to the original analysis.

### NASA-TLX

All questions of the modified NASA-TLX as described in the methods section were answered on a scale of 1 to 7. A separate ANOVA was carried out for each question. The main effect by noise on Mental Demand was significant, $F(3,69) = 6.277, p = 0.001, \eta ^{2}_{p} = 0.214$. Bonferroni-adjusted post-hoc tests showed significantly higher Mental Demand only when speech was presented compared to silence (5.37 vs. 4.08, $p = 0.006$). The effect by noise on Perceived Effort was also significant, $F(3,69) = 5.645, p = 0.002, \eta ^{2}_{p} = 0.197$. The Bonferroni-adjusted post-hoc tests revealed significantly higher Perceived Effort for speech (5.46) compared to silence (4.33, $p = 0.005$) and white noise (4.58, $p = 0.037$). The ANOVA on Task Difficulty revealed a significant effect by noise, $F(3,69) = 10.339, p < 0.001, \eta ^{2}_{p} = 0.310$. The Bonferroni-adjusted post-hoc tests showed significantly higher reported Task Difficulty for speech compared to silence (5.25 vs. 3.75, $p < 0.001$) as well as speech compared to white noise (5.25 vs. 4.21, $p = 0.001$). The main effect by noise on Performance was significant, $F(3,69) = 3.696, p < 0.016, \eta ^{2}_{p} = 0.138$. The Bonferroni-adjusted post-hoc tests showed significantly lower self-reported Performance for speech compared to white noise (4.54 vs. 5.29, $p = 0.031$). The ANOVAs ANOVA on self-reported Frustration and Well-being showed no significant differences.

**Fig. 9:**
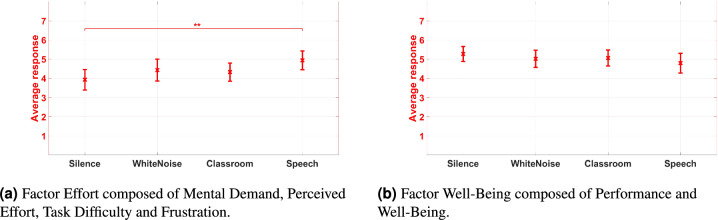
Results of factors extracted from extended NASA-TLX questionnaire regarding the introduced noise conditions. Significance levels are indicated by asterisks, where * = *p* < 0.05, ** = *p* < 0.01, *** = *p* < 0.001.

Since the questions on Mental Demand, Perceived Effort, and, Task Difficulty showed similar trends, redundancies between the items were investigated. For reliability analysis, Cronbach’s alpha was calculated. This assesses the internal consistency of the items. The internal consistency of the extended NASA-TLX questionnaire is questionable, with Cronbach’s $\alpha =.643$. Further, the questions regarding Mental Demand, Perceived Effort, Task Difficulty and Frustration were highly correlated, as well as the questions on Performance and Well-Being (see Table 2). Given these findings, it was hypothesized that the extended questionnaire might measure two separate constructs. A principal component analysis was considered. The Kaiser-Meyer-Olkin-criterium of .716 suggests that this analysis can be conducted. Also the Bartlett test was significant (p < .001). In accordance with the correlation analysis, the Scree plot and the explained variance suggested that the questionnaire contained two factors. The first factor explains 52.61% of the total variance and consists of questions Q1, Q2, Q3 and, Q5 and is further on called Effort. The second factor explains 24.52 % of the total variance and consists of questions Q4 and Q6 and is further on called Well-Being. A separate ANOVAwas carried out for each factor. The results are plotted in Fig. 9. The main effect by noise on Effort was significant, $F(3,69) = 5.637, p = 0.002, \eta ^{2}_{p} = 0.197$. Bonferroni-adjusted post-hoc tests indicate lower effort in silence compared to the classroom noise with speech (3.93 vs. 4.94, $p=0.009$). The main effect by noise on Well-Being was not significant, $F(3,69) = 1.708, p = 0.173, \eta ^{2}_{p} = 0.069$.

**Table 2:** Pearson-correlation of extended NASA-TLX questionnaire. MD: Mental Demand, PE: Perceived Effort, TaD: Task Difficulty, P: Performance, F: Frustration, WB: Well-Being. Significance levels are indicated by asterisks, where * = *p* < 0.05, ** = *p* < 0.01, *** = *p* < 0.001.

	MD	PE	TaD	P	F	WB
MD	1	$$0.763^{***}$$	$$0.786^{***}$$	− 0.192	$$0.635^{**}$$	− 0.118
PE		1	$$0.816^{**}$$	− 0.171	$$0.500^{*}$$	− 0.130
TaD			1	− 0.013	$$0.536^{**}$$	− 0.108
P				1	− 0.238	$$0.526^{**}$$
F					1	− 0.332
WB						1

### Slater-Usoh-Steed presence questionnaire

To evaluate the SUS, a separate ANOVA was conducted for each question. The effect of noise was significant for all questions. The results are stated in Table 3. If applicable, Bonferroni-adjusted post-hoc tests were performed.

**Table 3:** ANOVA results for Slater-Usoh-Steed questionnaire regarding the different noise types.

	*df*	*F*	*p*	$\eta ^{2}_{p}$
$\mathrm {SUS_{Q1}}$	(3,69)	8.387	<0.001	0.267
$\mathrm {SUS_{Q2}}$	(3,69)	9.095	<0.001	0.283
$\mathrm {SUS_{Q3}}$	(3,69)	7.773	<0.001	0.253
$\mathrm {SUS_{Q4}}$	(3,69)	5.280	0.002	0.187
$\mathrm {SUS_{Q5}}$	$(1.600,69)^{a}$	3.734	0.042	0.140
$\mathrm {SUS_{Q6}}$	(3,69)	8.008	<0.001	0.258

The full questionnaire including scales is given in the supplementary material. $^{a}$ Greenhouse-Geisser correction applied with an estimated $\epsilon _{gg} = 0.533$ due to significant in the sphericity test.

Question $\mathrm {SUS_{Q1}}$ indicated significantly lower sense of being in the classroom during silence (3.54) than classroom (4.88, $p = 0.001$) and speech (4.83, $p = 0.013$) noise. The reported sense of being there was also significantly higher for classroom compared to white noise (4.88 vs. 3.58, $p = 0.007$). According to $\mathrm {SUS_{Q2}}$, the feeling of the classroom being the reality was significantly stronger when the speech was presented compared to silence (4.67 vs. 2.96, $p = 0.008$), as well as classroom noise (4.25, $p = 0.017$) and speech (4.67, $p < 0.001$) compared to white noise. In line with this, question $\mathrm {SUS_{Q3}}$ indicated a significantly higher feeling of a visited place when classroom noise (4.58, $p = 0.004$) or speech (4.79, $p < 0.001$) were presented compared to white noise (3.29). Further, $\mathrm {SUS_{Q4}}$ revealed a significantly higher sense of being in the classroom when speech was presented (4.96) compared to silence (3.75, $p < 0.015$) and white noise (3.46, $p < 0.031$). $\mathrm {SUS_{Q5}}$ showed a tendency towards higher remembrance similar to other places when speech was presented compared to silence (4.73 vs. 3.79, $p = 0.061$). Finally, $\mathrm {SUS_{Q6}}$ revealed a higher feeling of really being in the classroom scenario when speech was presented compared to silence (4.42 vs. 3.21, $p = 0.021$), as well as for classroom noise (4.13, $p = 0.031$) and speech (4.42, $p = 0.007$) than white noise (3.04).

### Head movement

To track head movement, the HMD’s rotation and position, corrected for head center, were sampled at 5 Hz. For each participant, these positions were normalized relative to their initial head position at the start of the experiment. A brief evaluation of each noise condition revealed no rotation exceeding 7$^\circ$, with only minor differences between the noise conditions. Thus, no further analysis of the head rotation was conducted.

## Discussion

This study aimed to investigate the impact of realistic noise scenarios on ASA switching in a virtual classroom, focusing on task performance, perceived listening effort, and presence. We used a child-appropriate VR classroom paradigm, previously validated by Breuer et al.^10^, under conditions of silence, white noise, non-stationary classroom noise, and classroom noise with intelligible speech. The study included variables such as voluntary attention switch, stimulus congruence, and spatial target-distractor positions.

We hypothesized that switching auditory attention would be more demanding than maintaining focus on a single target position. This effect is only represented in the RTs. The paradigm, originally designed for children^9^, appears less challenging for adults. Since the overall trends remained, it is still useful for preliminary testing before involving children.

A congruence effect was expected, with better performance in congruent trials, which was confirmed by improved RTs and ERs. We also hypothesized that a larger spatial distance between target and distractor would improve performance, while front-back positioning would be most challenging due to binaural reproduction issues. Results confirmed that front-back positioning was indeed the most challenging due to front-back confusion and in-head localization with the generic HRTF. Despite using dynamic playback with head-tracking, participants rarely moved their heads, limiting the effectiveness of dynamic reproduction. The interaction between the target-distractor position-combination and attention transition as well as congruence in RTs was significant only for the front-back trials, highlighting the complexity introduced by binaural reproduction. In contrast, the interaction between congruence and target-distractor position-combination appeared to reflect only the main effect of congruence, regardless of the spatial positioning of the sound sources. This suggests that ERs may not be sensitive enough to detect behavioral changes in the paradigm for adults. Overall, task performance results in terms of RTs and ERs aligned with previous findings^5,7,9,10^.

The main goal of this study was to examine the impact of realistic noise on ASA as classified by the factors discussed above, hypothesizing that simple acoustic reproductions might underestimate the detrimental effect caused by noise.

An interaction between noise and congruence indicated lower RTs for white noise and classroom noise in congruent trials, suggesting that additional noise impairs task performance. This could be caused by processing of the noise and thus limiting cognitive resources or simply masking target signals. While these findings are partly in line with Yadav et al.^16^, especially the speech noise was expected to impair task performance even more severely. The absence of this interaction for speech noise could indicate an overall increase in task difficulty by the processing of the speech noise.

For ERs, the noise and congruence interaction showed higher ERs in incongruent trials for classroom and speech noise compared to silence, indicating increased task difficulty in these trials. This supports the hypothesis that simplified acoustic reproductions like white noise underestimate the impact of noise, while complex scenes increase task difficulty by limiting cognitive resources due to processing of irrelevant sound or attention capturing elements.

The interaction of attention transition and noise in RTs indicated a non-significant trend of switch costs under white noise only. This is mildly surprising, since white noise, in contrast to the other noise types, was not expected to increase task difficulty and thus make attention switches more challenging. This would be in line with the interaction of AT and C. However, since the interaction was non-significant, this trend needs to be treated with caution and should be investigated in more detail. It could, however, suggest a related effect for children. The same interaction showed a non-significant trend towards higher ERs in switch trials under speech noise, suggesting potential switching costs due to processing intelligible speech. Further, tendencies towards lower ERs in silence trials were found compared to speech trials under attention switches. Fewer errors were made in silence compared to classroom noise under repetition trials. These tendencies are similar to the interaction found for congruence (C). Contradictory to the non-significant trend in RTs, this would support the hypothesis that the realistic noise might affect adults’ performance, while white noise shows no effect^9^. All in all, more evidence for the interaction of more plausible noise and auditory attention switches in adults needs to be collected. Still, this effect might be more pronounced in children, who are generally more susceptible to noise^9,18^.

However, the study may still underestimate the impact of intelligible speech as a distractor, as the speech noise did not contain speech in every trial. To account for this limitation, a further exploratory analysis of only the trials containing speech was conducted. Here, the data of multiple participants had to be excluded given missing data points. Comparably to the main effect of noise, tendencies in RTs as well as a significant main effect in ERs indicated worse task performance in the speech-only and classroom-noise compared to the silence baseline. The expected difference between the two complex noise scenarios might be explained by the low number of trials and should be further investigated in a future study.

It remains unclear why the speech signals did not impair ASA more dramatically, since an effect of informational masking caused by the speech was expected to be strong additionally to the overall distraction caused by the noise^17^. Further, the speech signals used were recordings of normal and whispered speech, which is even more disruptive^65^. Further studies should look into the effect of each speech type and include longer utterances even addressing the participant directly.

Another shortcoming of the designed noise was a tendency towards more noise sources on the participants’ left side, since the windows were placed there and thus, outside noise in the classroom and speech conditions was introduced only to this side. An exploratory analysis showed no increased task difficulty for target positions on the left position, which could have been masked due to the increased amount of noise. Thus, the task is robust against the unequal distribution of noise sources, which again also reflects a realistic scenario, and the suggested classroom noise can be used and adapted for further studies.

Next to the task performance, questionnaires on perceived listening effort, as well as presence, were employed. This was expected to give insight into the demand introduced by the paradigm as well as the perceived realism introduced by the audiovisual virtual reality environment. It was hypothesized that a more complex noise would increase perceived listening effort as well as presence. Differences in the adapted NASA-TLX questionnaire were found between silence and speech for mental demand, perceived effort and task difficulty. These questions yielded higher ratings for speech than the silence baseline. These findings directly relate to the behavioral data reflected in the interaction effect of noise and congruence on ERs. Ratings for performance, perceived effort and task difficulty were also higher for the speech than the white noise condition. This indicates that adult participants felt more impaired by the speech than white noise, although a respective effect cannot be seen in the behavioral data. Given that the principal component analysis performed on the extended NASA-TLX questionnaire suggested to reduce the questionnaire to two factors and the resulting factor of Effort only showed increased effort for speech compared to silence, the difference between speech and white noise can be neglected. In future studies, it should be considered to use only a subset of the NASA-TLX questions, e.g., the proposed factors of Effort and Well-Being. This reduced approach to measure listening effort has been successfully applied in a recent study by Seitz et al.^66^.

Participants also reported higher difficulty in speech trials given a masking of the cue and target sound by the disturbing speech. It is interesting to note that the non-stationary spatially distributed classroom sound did not yield any perceived increased listening effort, although the behavioral data indicate otherwise. This is partially in line with the findings by Leist et al.^18^ who found even better performance in a listening comprehension task for binaural as opposed to monaural noise, possibly due to an increased SNR introduced by spatial release from masking. While noise including semantic content is associated with increased listening effort^67^, Villard et al.^68^ found that different masker types elicited different physiological responses. This was shown in a study investigating the impact of energetic and informational masking on listening effort measured by electroencephalogram (EEG) and pupillometry. Thus, including such measures could be beneficial to get further insight into how the listening effort induced by the classroom noise with and without speech might influence the ASA.

Regarding the SUS presence questionnaire, presence was rated highest in the speech condition, which was the expected behavior. A similar amount of presence was introduced by the classroom sound. The presence in the silence condition was rated lower than the two realistic noise scenes. Surprisingly, the white noise showed the least amount of perceived presence, which indicates that an unrealistic acoustic scenario might impair the presence in an audiovisual virtual reality environment even more severely than introducing no background sound at all.

Still, using a questionnaire gives only insight into the perception of the virtual environment, but limited information on the audiovisual integration. It would, therefore, be interesting to investigate the relation between enhanced presence, motivation, and task performance. Thus, further studies could put more focus on this topic by also investigating plausibility and immersion. To get further insight into the impact of the virtual environment as well as to entangle the impact of visual and acoustic scenes on ASA, extended measures such as collecting EEG data^28,69^ could also be helpful.

Finally, head movement as a measure of engagement in the virtual classroom was tracked but did not seem to be an informative measure for this study. The data showed very little movement which could be due to the purely auditory task as well as the display of all relevant information such as instructions and feedback on a blackboard in front of the participants, which was already discussed in a previous study by Breuer et al.^10^. Therefore, it was not further analyzed. Adding visual representations of the target and noise stimuli in the form of virtual agents might encourage participants to move their heads and explore the VR environment. This extension needs to be the next step to increase the realism of the proposed design.

In summary, the present study introduced a child-appropriate VR paradigm situated in a virtual classroom environment and investigated the impact of silence, white noise and non-stationary ambient classroom sounds. For now, the study was only conducted with adult participants to validate the adapted paradigm and create a baseline for future studies including children. While the acoustic setting was designed with children in mind, the inclusion of typical sounds such as chair movements and pen scraping resulted in a scenario that may also resemble open-plan office environments, potentially enhancing its relevance for adult participants apart from teachers. Further, the participant group consisted mainly of university students who only recently left the school environment and still encounter similar situations during smaller lectures and seminars (compare the virtual seminar room used by Schiller et al.^70^ and Ehret et al.^71^). However, to fully understand how children respond to such environments, it will be essential to conduct follow-up studies with child participants. This represents not only a limitation of the current study but also an important opportunity to extend the paradigm and explore developmental aspects in closer-to real-life, immersive contexts.

The proposed study underlines the importance of creating more plausible research scenarios by introducing not only visual representations of everyday situations but also including complex acoustic scenes, since simplified acoustic representation might underestimate the noise effect people are faced with in their everyday lives, which is expected to be even more severe for children than adults.

## Supplementary Information

Supplementary Information.

## Data Availability

The software used to conduct the presented study, as well as the collected data are publicly available in “Impact of Realistic Noise Scenarios on Auditory Selective Attention Switch in a Virtual Classroom Environment” at https://doi.org/10.5281/zenodo.12688235 under the Creative Commons Attribution 4.0 International license. The acoustic stimuli for the task are taken from Loh and Fels 2023 “ChildASA dataset: Speech and Noise Material fpr Child-appropriate Paradigms on Auditory Selective Attention” at https://doi.org/10.18154/RWTH-2023-00740 under the Creative Commons Attribution-NonCommercial-NoDerivs 3.0 Germany (CC BY-NC-ND 3.0DE) license.
