# Supplementary material for: The influence of complex classroom noise on auditory selective attention
Source: Sci Rep. 2025 Sep 25;15:32926. doi: 10.1038/s41598-025-18232-2 (PMC12464317; doi:10.1038/s41598-025-18232-2)
Supplement: Supplementary file 1 — Supplementary Information. [file 41598_2025_18232_MOESM1_ESM.pdf]

1 Supplementary Methods

Questionnaires

Listening Effort

Listening effort was measured by an adapted and exptended version of the NASA-Task Load Index with the dimensions Mental Demand, Perceived Effort, Task Difficulty, Frustration, Performance, and Well-Being[1,2,3,4]. All questions were answered on a scale from 1 (very low) to 7 (very high). The questions were:

1. Wie geistig anstrengend war die Aufgabe?
   Engl.: How mentally demanding was the task?

2. Wie hart mussten Sie arbeiten, um Ihren Grad an Aufgabenerfüllung zu erreichen?
   Engl.: How hard did you have to work to achieve your level of task performance?

3. Wie schwierig war die Aufgabe?
   Engl.: How difficult was the task?

4. Wie erfolgreich waren Sie Ihrer Meinung nach beim Erfüllen der Aufgabe?
   Engl.: How successful do you think you were in completing the task?

5. Wie unsicher, entmutigt, irritiert, gestresst und verärgert fühlten Sie sich während der Aufgabe?
   Engl.: How insecure, discouraged, irritated, stressed and annoyed did you feel during the task?

6. Wie gut haben Sie sich beim Bearbeiten der Aufgabe gefühlt?
   Engl.: How good did you feel while performing the task?

Presence

To measure presence in the virtual reality environment, a German version of the Slater-Usoh-Steed (SUS) presence questionnaire[5] was used. All questions were rated on a 7-point scale and are stated below.

SUS_{Q1} Ich hatte das Gefühl, ‚da zu sein‘ in der Szene. (1) Überhaupt nicht. (7) Sehr.
Engl.: I had a sense of ’being there’ in the scene. (1) Not at all. (7) Very much.

SUS_{Q2} Zu welchem Ausmaß gab es Momente, in denen die Gesprächssituation die Realität für Sie war? (1) Nie. (7) Fast immer.
Engl.:To what extent were there times during the experience when the conversation was the reality for you? (1) At no time. (7) Almost all the time.

SUS_{Q3} Wenn Sie an die Erfahrung zurückdenken, stellen Sie sich die Gesprächssituation eher vor als Bilder, die Sie gesehen haben, oder als Orte, die Sie besucht haben? (1) Bilder gesehen. (7) Orte besucht.
Engl.: When you think back about your experience, do you think of the conversation more as images that you saw, or more as somewhere that you visited? (1) Images that I saw. (7) Somewhere that I visited.

SUS_{Q4} Während der Erfahrung, was war insgesamt am stärksten, Ihr Gefühl, in dem Klassenraum zu sein, oder sich woanders zu befinden? (1) Woanders. (7) Im Klassenraum.
Engl.: During the time of the experience, which was strongest on the whole, your sense of being in the classroom, or of being elsewhere? (1) Being elsewhere. (7) Being in the classroom.

SUS_{Q5} Ich empfinde die Gesprächssituation als Ort ähnlich zu anderen Orten, an denen ich heute war... (1) Überhaupt nicht. (7) Sehr.
Engl.: I think of the conversation as a place in a way similar to other places that I’ve been today... (1) Not at all. (7) Very much so.

SUS_{Q6} Während der Erfahrung dachte ich oft, dass ich tatsächlich in der Gesprächssituation bin... (1) Nicht sehr oft. (7) Sehr oft.
Engl.: During the experience I often thought that I was really standing in the conversation... (1) Not very often. (7) Very much so.

References

1. Hart, S. G. Nasa-task load index (nasa-tlx); 20 years later. *Proceedings of the Human Factors and Ergonomics Society Annual Meeting* **50**, 904–908 (2006). URL https://doi.org/10.1177/154193120605000909. https://doi.org/10.1177/154193120605000909.
2. Mackersie, C. L. & Cones, H. Subjective and psychophysiological indexes of listening effort in a competing-talker task. *Journal of the American Academy of Audiology* **22**, 113–122 (2011). https://doi.org/10.3766/jaaa.22.2.6.
3. Bologna, W. J., Chatterjee, M. & Dubno, J. R. Perceived listening effort for a tonal task with contralateral competing signals. *The Journal of the Acoustical Society of America* **134**, EL352–EL358 (2013). URL https://doi.org/10.1121/1.4820808. https://pubs.aip.org/asa/jasa/article-pdf/134/4/EL352/15310439/el352_1_online.pdf.
4. Francis, A. L. & Love, J. Listening effort: Are we measuring cognition or affect, or both? *WIREs Cognitive Science* **11**, e1514 (2020). URL https://wires.onlinelibrary.wiley.com/doi/abs/10.1002/wcs.1514. https://wires.onlinelibrary.wiley.com/doi/pdf/10.1002/wcs.1514.
5. Usoh, M., Catena, E., Arman, S. & Slater, M. Using presence questionnaires in reality. *Presence* **9**, 497–503 (2000).
